# Symptomatic inguinal bladder hernia causes post-renal acute kidney injury: A rare case report

**DOI:** 10.1016/j.amsu.2020.09.033

**Published:** 2020-09-28

**Authors:** Nasam Alfraji, Steven Douedi, Mohammad Hossain

**Affiliations:** a Department of Medicine, Jersey Shore University Medical Center, Neptune, NJ, 07753, USA

**Keywords:** Inguinal hernia, Bladder hernia, AKI, Surgery, Urinary symptoms, Case report

## Abstract

**Introduction:**

Inguinal bladder hernia (IBH) is a rare condition representing less than 5% of all inguinal hernias. Most cases occur in elderly overweight men. Patients may present with variable symptoms such as urinary symptoms, inguinal swelling, or pain; however, most of them are asymptomatic and only less than 7% are diagnosed pre-operatively. Different radiological studies can be used if IBH suspected preoperatively including ultrasound, computed tomography scan; however, cystography is the most sensitive test for diagnosis of IBH. Open reduction and hernia repair are the standard treatment of IBH.

**Case presentation:**

We report a rare case of an-83-year-old male who presented with left inguinal pain associated with lower urinary tract symptoms including dysuria, nocturia, post-voidal dribbling, and urinary frequency. Laboratory studies showed acute kidney injury (AKI), and computed tomography (CT) of abdomen and pelvis without contrast CT revealed a herniation of 80% of the bladder through the left inguinal canal into the left scrotal sac, with moderate bilateral hydronephrosis and hydroureter, though no obstructing calculi are seen. Pre-operative diagnosis of incarcerated inguinal bladder hernia (IBH) was made, and a timely surgical intervention preceded by bladder catheterization led to a significant improvement of AKI and an excellent outcome without post-operative complications.

**Discussion and conclusion:**

IBH is uncommon condition that can present with non-specific urinary symptoms; therefore, high index of suspicion is mandated for diagnosis especially in patients with risk factors. Pre-operative radiological evaluation to avoid iatrogenic bladder injury with subsequent surgical repair is the standard management for IBH as we accomplished in our case.

## Highlights

• Consider inguinal bladder hernia in patients with urinary symptoms and large inguinal hernia.
• Proper evaluation and pre-operative diagnosis of inguinal bladder hernia is important to prevent any iatrogenic bladder injury.
• Pre-operative urinary catheterization and surgical repair is the treatment of choice in inguinal bladder hernia.

## 1 Introduction

Inguinal bladder hernia is a rare entity which represents only 1–5% of all inguinal hernias [2]. Main factors disposing to this condition including male gender, advanced age, obesity, bladder obstruction, abdominal wall weakness or diseases [3]. Most commonly, bladder hernias are seen on the right side, and usually diagnosed incidentally through imaging or during surgery with only less than 7% diagnosed pre-operatively [[2], [3], [4]]. Bladder catheterization is recommended to exclude and/or relieve any urinary retention, and surgical repair is the treatment of goal [2]. We report an-83-year-old male presented left groin pain with lower urinary symptoms and found to have a post-renal acute kidney injury in the setting of a left incarcerated inguinal bladder hernia. This case has been reported in line with the SCARE criteria [1].

## 2 Case presentation

An 83-year-old male with a history of prostate cancer status post transurethral resection of the prostate (TURP), diabetes type 2 on insulin, and hypertension presented to the emergency department (ED) complaining of left inguinal pain for several days associated with worsening dysuria for the past month. He stated he has had increased size of his scrotum during this time and has been having post void dribbling, urinary frequency, and nocturia. He stated he visited a urologist about one year prior to presentation who told him he had bilateral hydroceles found on scrotal ultrasound. He had no other complains on admission and was in no apparent distress. Family history was significant for hypertension and coronary artery disease in mother. He is a former smoker and drinks alcohol socially. Drug history includes amlodipine, carvedilol, furosemide, ramipril, atorvastatin, insulin, tamsulosin, and oxybutynin.

In the ED, vital signs were unremarkable with a temperature of 99.0° Fahrenheit, blood pressure of 131/61 mm Hg, heart rate of 82 beats per minute, and oxygen saturation of 96% on room air. His body mass index (BMI) was 30. Physical examination revealed bilateral enlargement of the scrotum in particular the left hemi-scrotum. There were no skin changes, no erythema and nontender. The findings were consistent with a chronically incarcerated but not strangulated inguinal scrotal hernia on the left. The rest of physical examination was unremarkable.

**Fig. 1:**
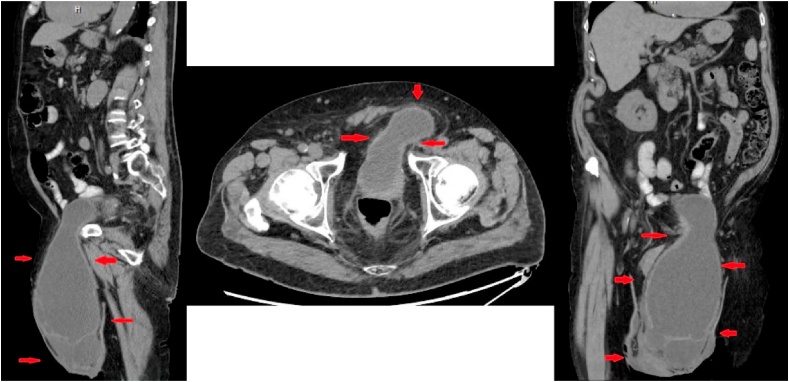
CT abdomen and pelvis without contrast revealing a herniation of 80% of the bladder through the left inguinal canal into the left scrotal sac.

Laboratory testing was significant for a white blood cell count of 12.3 10*3/μL (normal value: 4.5–11 10*3/μL), blood urea nitrogen of 93 mg/dL (normal value: 5–25 mg/dL), and creatinine of 3.34 mg/dL (normal value: 0.61–1/24 mg/dL) (Table 1). A urine analysis with turbid appearance, yellow in color, too numerous to count white blood cells, moderate leukocytes, many bacteria, and negative nitrites. Scrotal ultrasound with doppler study showed a left sided moderate to large complex hydrocele containing debris and a moderate to large right sided hydrocele. A computed tomography (CT) scan of the abdomen and pelvis revealed a herniation of the bladder approximately 80% through the left inguinal canal into the left scrotal sac with the portion of the bladder containing the uretero-vesical junction remains within the pelvic cavity (Fig. 1). Moderate bilateral hydronephrosis and hydroureter were noted, but no obstructing calculi were seen.

**Table 1:** Summary of main laboratory investigations at baseline and during admission.

Lab	Baseline value	At admission	After intervention	Reference value
Hemoglobin	13.5	10.7	9.6	12–16 g/dL
WBC	6	12.3	6.2	4.5–11.0 K/uL
Bands	–	28.0	–	5–11%
Platelets	233	312	160	140–450 K/uL
BUN	22	93	31	5–25 mg/dL
Creatinine	1.05	3.34	1.5	0.61–1.24 mg/dL
GFR	>60	18	42	>60 ml/min/1.73
Sodium	140	135	138	136–145 mmol/L
Potassium	3.8	4.8	3.8	3.5–5.2 mmol/L
Calcium	9	10	9.4	8.5–10.5 mg/dL

A foley catheter was placed and he was started empirically on ceftriaxone 1 g every 24 hours for his complicated urinary tract infection. Urology, surgery and infectious disease were consulted for further management. On day 2, urine culture grew *Staphylococcus caprae* and blood cultures remained without growth. His antibiotics were switched to cefazolin 1 g twice daily for a total of 2 weeks of antibiotic treatment. The decision was also made for elective surgical intervention for his bladder herniation. On day 6, he underwent surgical left inguinal hernia reduction and repair with phasix mesh placement without complications using open tension-free mesh repair technique. Surgery was performed under general anesthesia primarily by a general surgeon who has more than 30 years of experience with the assistance of a fourth-year surgical resident. The patient tolerated the surgery very well and remained with an uncomplicated post-operative course. His symptoms and kidney function improved significantly afterward.

The patient was discharged to subacute rehab with an indwelling foley catheter on day 8 of hospitalization and he had his foley catheter removed within one week of discharge with a successful voiding trial. He reported no post-operative complications such as pain or recurrence at his latest follow-up visit.

## 3 Discussion

Inguinal hernia is the most common type among the abdominal wall hernias and accounts for 70–75% of cases [5]. However, IBH is considered uncommon variety that constitutes 1–5% of all inguinal hernias [2]. Inguinal bladder hernia mentioned first by levine in 1951 as a rare variant of inguinal hernia [2]. Patients with inguinal bladder hernia are usually asymptomatic and most of them diagnosed intraoperatively in 77% of cases, less common post-operatively in 16%, and rarest pre-operatively in less than 7% [6]. However, IBH still can be symptomatic due to bladder obstruction or superimposed infection [3]. Patients may present with groin pain/swelling, worsening kidney function, urinary retention, lower urinary tract symptoms including dysuria, frequency, and nocturia [3,4]. Long-standing, un-repaired IBH can be complicated by renal failure, bladder perforation, recurrent stones, or hydroureteronephrosis [3]. Urological malignancies have been noted in 11.2% of patients with IBH in one of the studies in 2004 [6].

Diagnosis can be facilitated by using different imaging modalities including ultrasound, computed tomography (CT) scan, or magnetic resonance; however, cystourethrography remains the gold standard for diagnosis of IBH [3,7]. Surgical repair is the treatment of choice; however, pre-operative bladder catheterization is required to relieve any urinary retention and to reduce the hernia size [6]. The rate of bladder injury during surgery is still considered high at 12%, and it is lower if IBH is diagnosed pre-operatively [6].

Literature review revealed few similar reported cases that presented with urinary symptoms, urinary retention, bilateral hydronephrosis, acute kidney injury, or inguinal pain/swelling [4]. In most cases, hernia repair was the ultimate treatment for IBH [[2], [3], [4],^6^].

Our case presented with lower urinary tract symptoms associated with left inguinal pain who found to have incarcerated left inguinal bladder hernia on CT scan complicated by bilateral hydronephrosis and AKI. Elective surgical repair was performed successfully, without any post-operative complications. Patient had short recovery post-operatively with a significant improvement of his kidney function and his symptoms.

## 4 Conclusion

IBH is a rare and under-recognized pathology which is more common in high risk population such as elderly, overweight men. Clinical presentation is variable including non-specific urinary symptoms, inguinal pain/swelling, or even asymptomatic in most cases; therefore, high index of suspicion is required for diagnosis. Our case emphasizes the importance of proper pre-operative evaluation including imaging and the timely surgical repair to reverse any established complications and prevent any further ones as demonstrated by our case. Pre-operative or intra-operative exclusion of urological malignancy is also recommended as literature showed some association between the IBH and most commonly bladder cancer.

## Provenance and peer review

Not commissioned, externally peer reviewed.

## Declaration of competing interest

The authors declare that they have no competing interests.

## Registration of research studies

Name of the registry: n/a.

Unique Identifying number or registration ID: n/a.

Hyperlink to your specific registration (must be publicly accessible and will be checked): n/a.
